# Impact of Nutritional Strategies to Prevent Post-Weaning Diarrhoea on Performance, Behaviour, and Microbiota in Piglets from Organic Farming

**DOI:** 10.3390/ani14121730

**Published:** 2024-06-08

**Authors:** Marc Bagaria, Yuliaxis Ramayo-Caldas, Olga González-Rodríguez, Lluís Vila, Pino Delàs, Emma Fàbrega

**Affiliations:** 1 Animal Welfare Program, Institute of Agrifood Research and Technology (IRTA), 17121 Monells, Spain; emma.fabrega@irta.cat; 2 Animal Breeding and Genetics Program, Institute of Agrifood Research and Technology (IRTA), 08140 Caldes de Montbui, Spain; yuliaxis.ramayo@irta.cat (Y.R.-C.); olga.gonzalez@irta.cat (O.G.-R.); 3 Llavora Agropecuària, 17473 Ventalló, Spain; lluis@saiga.es (L.V.); pinodelas@gmail.com (P.D.)

**Keywords:** post-weaning diarrhoea, behaviour, organic, piglet, low-protein diet, whey, performance, microbiota

## Abstract

**Simple Summary:**

Organic pig production aims to ensure high animal health and welfare standards. Despite this, diseases such as post-weaning diarrhoea may affect piglets after weaning. Given that the use of drugs is limited on organic farms, other prevention strategies, like nutritional interventions, have become a promising approach to preventing post-weaning diarrhoea. In this study, we fed the animals with three diets: high crude protein feed, low crude protein feed, and low crude protein feed supplemented with liquid whey. These diets were given to the piglets four weeks after they were weaned, and their diarrhoea incidence, health, behaviour, and intestinal microbiota composition were assessed. The results of this study showed that the diet did not affect diarrhoea incidence. However, the piglets receiving the diet supplemented with liquid whey showed increased growth as well as changes in their behavioural patterns, such as a lower percentage of negative social behaviours and drinking. In addition, there was an increase in beneficial bacteria in the intestinal microbiota of piglets fed liquid whey. These results altogether indicate that liquid whey could be a valuable supplement for pigs in organic farming, showing positive effects on some behaviour patterns, microbiota composition, and performance efficiency.

**Abstract:**

Organic livestock farming is committed to high environmental and animal welfare standards, although pathologies such as post-weaning diarrhoea (PWD) may appear. The main objective of this study was to assess nutritional strategies to prevent PWD in organic piglets. A total of 134 weaned piglets were fed one of three diets: high crude protein (17.8%, HCP), low crude protein (16.8%, LCP), and low crude protein supplemented with liquid whey (LCP+W). Piglets were assessed weekly for four weeks on the following parameters: diarrhoea incidence, additional health parameters, average daily gain, and behaviour. Faecal samples were taken to analyse the intestinal microbiota composition. Data were analysed using LMM and GLMM models and Shannon and Whittaker indexes. No significant effect of diet on diarrhoea incidence was found, but the LCP+W diet increased average daily gain. Pigs fed the LCP+W diet presented a lower percentage of drinking and negative social behaviour compared with the HCP diet, and LCP pigs presented higher exploration compared with HCP. In addition, LCP+W piglets showed a higher abundance of the beneficial genus *Frisingicoccus*. Although liquid whey did not reduce diarrhoea incidence, the benefits found in growth, microbiota composition, and reduced negative social behaviour indicate that it could be an optimal supplement to organic diets.

## 1. Introduction

Organic livestock farming is a production system with a high potential to allow an enhanced expression of the behavioural repertoire in many species and, thus, promote higher animal welfare standards [1]. Moreover, organic production in the EU is regulated by Regulation (EU) 2018/848 [2], which limits housing conditions, animal breeding, nutrition, disease prevention, and veterinary control. However, organic livestock husbandry does not always ensure higher health standards for the animals compared with conventional farming, despite its demonstrated benefits to the environment and animal welfare [3].

Post-weaning diarrhoea (PWD) is one of the main health concerns in pig farming worldwide, especially because the use of prevention strategies like zinc oxide or antimicrobials has increasingly been limited or banned [4]. This syndrome consists of the deposition of watery faeces that usually starts 3 or 4 days post-weaning and can last for two weeks [5]. This enteric disease is mostly caused by the proliferation of the enterotoxigenic *Escherichia coli* (ETEC) phenotype in the piglet small intestine [6]. Piglets suffering from diarrhoea present a vast amount of ETEC in their gut, finally resulting in dehydration, diarrhoea, decreased nutrient digestibility, behavioural changes, a reduced growth rate, and even the death of the animal [4]. PWD is a multifactorial syndrome related to the physiological, social, and environmental stressors associated with the weaning process. This process consists of the physical separation of the piglet from their mother, with a consequent change in the piglets’ diet from liquid maternal milk to solid feedstuffs [7]. These diet changes cause a major transformation in the gastrointestinal immunology, physiology, and microbiota of weanling piglets, which makes them more susceptible to *Escherichia coli* infections [8]. The weaning process also requires important behavioural adaptations in the piglets since they are usually moved to a new environment where a new social structure is created. As a result, stress-related behaviours appear, such as less time eating and drinking that result in a reduction in voluntary feed and water intake [9], as well as other detrimental behaviours, such as tail biting, which have been found to be at the highest incidence in weaners [10].

Given that the use of medicines is not allowed as a preventive measure in organic farming [2], farmers need to apply animal husbandry preventive strategies aimed at avoiding or minimising the development of certain diseases, such as PWD [1]. Some strategies that have been suggested are the avoidance of mixing litters, adequate hygiene, optimal temperature and ventilation, a low stocking density, and providing the animals with an appropriate diet that is accessible to all the piglets [4]. Along that line, a reduction in the crude protein (CP) content of the feed is a method that has been widely studied and implemented in pig production in order to reduce environmental impacts and has also been found to have a beneficial impact on diarrhoea incidence if the amino acid content is properly balanced [11].

Diets with high values of crude protein cause maldigestion because the digestive system of piglets is not fully developed [12], encouraging the proliferation of pathogens such as ETEC that promote the development of PWD [13]. Previous studies showed that decreasing the amount of crude protein from 210–250 g/kg to less than 170 g/kg reduced the incidence of PWD [14,15,16] compared with diets with a higher content of crude protein and also decreased the number of ETEC in the intestine [17]. Despite the advantages that a diet with low crude protein content presents, farmers are reluctant to use this nutritional approach because piglets fed this diet may show a decreased growth rate compared with piglets receiving a diet with a higher protein content [15,16,18]. Moreover, some studies have indicated that pigs receiving a low CP diet (13.7%) showed more ear and tail biting, belly nosing, and other oral manipulation directed at pen mates and performed more aggression than pigs receiving a control CP diet (16.8%) [19]. These effects can be reversed by supplementing the low-protein diet with alternative protein sources and balancing the amino acid content. In organic farming, the legal limitations on the feed ingredients restrict an optimal protein supply, and diets require to be balanced accordingly, either with organic plant-based sources such as soybean, sunflower, rapeseed, and grain legumes or other potential ingredients such as whey from organic sources [20].

Whey is a liquid by-product of the cheese industry that is obtained in vast quantities during the curdling phase of cheese production [21]. Moreover, whey is one of the most polluting by-products that exist in the food industry and creates serious environmental problems because of its high content of organic matter; thus, alternatives should be explored to decrease the impact of this waste product [22]. Whey is a valuable feedstuff because it contains a high amount of lactose and proteins and is a good source of calcium, phosphorus, sulphur, chlorine, and other minerals, as well as group B vitamins and ascorbic acid [21]. Some studies demonstrated that the use of whey as a supplement to the diet enhanced the piglets’ average daily weight gain and daily feed intake [23], while other studies showed that lactose improved the piglets’ health [24] and reduced the incidence and severity of PWD [25]. The effect of lactose on piglets’ health can be explained because it decreases the presence of potential pathogens, such as *E. coli* [26], while stimulating the presence of beneficial bacteria, such as bifidobacteria and lactobacilli [24,26].

The main objective of this study was to assess if low-protein diets, either alone or supplemented with whey, would prevent PWD in organic piglets without compromising their growth rates or having any negative impact on behaviour and welfare. To do so, three different diets were used: a control high crude protein feed (HCP), a low crude protein content feed (LCP), and a low crude protein content feed supplemented with liquid whey (LCP+W). Due to the availability of organic protein sources, the crude protein reduction implemented in this study was from 17.8% in the HCP to 16.8% in the LCP diet, which is a smaller reduction than in other studies conducted on conventional farms [13,14,15,16,27]. The hypothesis of this study was that the LCP diet would reduce PWD but also decrease the growth rate of piglets, and the LCP+W diet would reduce PWD without decreasing the growth rate compared with the HCP diet.

## 2. Materials and Methods

### 2.1. Animals and Husbandry

This study was conducted at the Llavora organic pig farm (Ventalló, Catalonia, Spain). The farm consisted of a closed-cycle unit including 34 sows, 1 boar, 70 weaned piglets, and 220 finishing pigs of *Swabian-Hall* and *Duroc* breeds. The sows farrowed 9 to 10 piglets per litter, and the piglets remained with the sow and their littermates until weaning at approximately 49 days of age. Given neonatal mortality, an average of 7 piglets were weaned per sow, and at weaning age, the sow was separated from the piglets, moving the sow to a different pen and leaving the piglets in the same pen. Weaned piglets were housed in a 13.75 m^2^ pen with an indoor and outdoor area. The outdoor run consisted of a 2.5 × 2.5 m (length × width) area with an earth floor and a drinking supply. The indoor area consisted of a 2.5 × 3 m area with a concrete floor provided with straw, a feeder, and a wooden nest with a red-light lamp. Water and feed were supplied ad libitum. The weaned piglets stayed in the pen from weaning until they were moved to the fattening phase. The ventilation system was natural, and the farm was not equipped with a system for automatic temperature control.

### 2.2. Experimental Design

The experiment was carried out from February (8.8 °C mean environmental temperature in the area) to July (23.8 °C mean environmental temperature in the area); thus, different seasons were evaluated. During this period, we studied a total of 134 piglets divided into five batches during their post-weaning period. On the weaning day, each animal in the batch was ear-tagged with an individual ID number. The weaned piglets were separated into three pens, each receiving one of the three diets: high crude protein feed (HCP), low crude protein feed (LCP), and low crude protein feed supplemented with liquid whey (LCP+W). Depending on the number of weaned piglets in each batch, the groups ranged from 5 to 14 piglets per pen, always balancing the number of individuals in the pens within a batch. The groups in each batch were formed by balancing the animals according to their weight, litter of origin, and sex (male or female) within a pen. All the animals in each batch were evaluated once a week for four weeks until they were moved to the fattening pens.

### 2.3. Diets

Both HCP and LCP diets were supplied ad libitum in the feeder situated in the inside area of the pen. These diets consisted of solid feeds made from mostly plant-based organic products containing a mix of wheat, barley, peas, oats, corn, soybean flour, and soybean oil in different proportions. The HCP feed consisted of a pre-starter feed that also contained a small proportion of milk powder coming from organic production. The LCP+W diet consisted of the same LCP feed supplemented with liquid whey that was supplied ad libitum in a second feeder situated in the inside area of the pen. Liquid whey was obtained twice a week from Mas Alba, a local cheese factory where fresh and cured cheese was produced. Thus, the type of whey produced and used in this study was acid whey with a pH of 3.9 and contained 4.07% p/p lactose, 1.13% p/p fat, 0.87% p/p protein, 0.51% ashes, 0.10% salt, and 0.04% sodium. The nutritional composition of the three diets is detailed in Table 1.

### 2.4. Measurements and Parameters Analysed

During the period the animals were in the weaning pens, they were assessed once a week, assessing each batch at four different time points: weeks 1, 2, 3, and 4. The first week corresponded to the weaning week, and the last week was the week the piglets were moved to the fattening phase. The parameters assessed were diarrhoea at individual and pen levels, additional health parameters, initial and final weight, feed and whey intake, and behaviour. In addition, gut microbiota composition was studied.

On each of the weekly evaluations, individual and group diarrhoea scores were noted. Any individual that presented visible diarrhoea during the behavioural observations or the individual examination was scored. The scores for diarrhoea at individual level were as follows: 0 = no diarrhoea: formed faeces; 1 = mild diarrhoea: pasty faeces; 2 = severe diarrhoea: liquid/watery faeces [29]. Before the behavioural observations, diarrhoea at the pen level was assessed, scoring the state of the visible and fresh dung in pen: 0 = no liquid manure visible; 1 = some liquid manure visible; 2 = all faeces visible are liquid manure [30]. If an outbreak appeared in between weekly recordings, the farmers were notified so that the researchers could proceed to score and register it.

In addition to diarrhoea, additional health parameters and behaviours were assessed based on the welfare quality assessment protocol for pigs [30]. Once we entered the facilities, respiratory health parameters were recorded at the group level, scoring for 5 min each time a piglet presented coughing, sneezing, or pumping. After these 5 min, the thermal comfort parameters were recorded at pen level, scoring the number of animals presenting huddling, shivering, or panting in the pen. Then, the behavioural observations at the pen level were conducted, and once they were finished, the observers entered the pens to assess the health status of the piglets at the individual level. All the animals were individually examined to assess their body condition score, skin condition, tail-biting lesions, and manure on the body (Table 2). Mortality was also recorded and calculated for the whole observation period.

On the first and last day of the weaning phase (evaluations weeks 1 and 4), all the piglets were individually weighed using a weighing beam set (MS Schippers, Granollers, Spain), obtaining initial and final weight measures. From these measures of individual body weight, the average daily gain (ADG) was calculated and expressed in grammes gained per day. The amount of solid feed the piglets consumed for the three different diets was measured by weighing the feed supplied to each of the pens during the four weeks. Then, feed intake was estimated and expressed as the average of kilos of feed consumed per individual during the weaning period. The total liquid whey consumed in the LCP+W diet was also measured, and the whey intake was estimated and expressed as the average of litres of whey consumed per individual during the weaning period.

For the behavioural observations, all the groups were observed using instantaneous scans and continuous sampling [31]. For the scan sampling, the behaviour of each of the individuals in the pen at that instant was recorded. Scan behaviours included positive social behaviour, negative social behaviour, pen investigation, exploring enrichment, resting, and others (Table 3). For the scan sampling, a total of 12 scans per pen were performed on each evaluation, one at the beginning of the observation and one after each continuous sampling, and were expressed as the proportion of the behaviour per total scan. For the continuous sampling, the particular behaviours of all the individuals in pen during 5 min were recorded. Continuous sampling behaviours included positive social behaviour, negative social behaviour, exploring enrichment, eating, drinking, and tail or ear biting (Table 3). A total of four continuous samplings per pen of 5 min each was performed on each evaluation and were expressed as the proportion of the behaviour per total minutes of observation. The observations were performed between 9:00 and 12:00 h by two trained observers at the same time, one situated in the outdoor area and the other in the indoor area, due to having a clear vision of all the individuals in the pen. The observers alternated the observation area inside and outside to balance the observations between treatment pens, and inter-observer reliability was calculated using a Kappa coefficient test to ensure both observers coded the behaviour similarly. The result of the Kappa coefficient test was 0.86, which indicated almost perfect agreement [31].

### 2.5. Microbiota Analysis

Individual faecal samples were obtained for most of the individuals in batch 5 following rectal stimulation on the last evaluation day (week 4). The 29 faecal samples obtained were immediately frozen at −80 °C and sent to the laboratory for analysis of the intestinal microbiota composition. Total DNA was extracted from 250 mg of each faecal sample using the DNeasy PowerSoil Kit (Qiagen, Hilden, Germany) according to the manufacturer’s instructions. DNA concentration and purity were checked with a NanoDrop ND-1000 spectrophotometer (Thermo Fisher Scientific, Waltham, MA, USA). Microbial profiling of the extracted DNA was performed using pair-end (2 × 250 nt) sequencing on an Illumina Miseq (Illumina, San Diego, CA, USA). The primers V3 and V4 were used to amplify the 16s rRNA gene fragment. Quality control was performed on the resulting FastQ files using FastQC software v0.12.0 [32], bioinformatics analyses were conducted using the Qiime2 package v2024.2 [33], and low-quality reads, primers, and barcode sequences were removed. The remaining sequences were processed into Amplicon Sequence Variants (ASVs) at 99% identity. ASVs representing less than 0.001% of the total counts and present in less than two samples were excluded, as well as samples with less than 10.000 reads. ASVs were classified to the lowest possible taxonomic level based on a primer-specific version of the GreenGenes2 Database [34].

### 2.6. Statistical Analysis

The assessed parameters were analysed using the software Rstudio, version 4.2.1 [35]. Tests were two-sided with an alpha value of 0.05. To conduct the statistical analysis of the assessed parameters, all 134 individuals studied were included.

The evolution of the diarrhoea scores over time was studied both at the individual and pen levels (i.e., the experimental unit was either individual or pen depending on evaluation) using an ordered logistic regression from the nnet and MASS packages v7.2-60.2 [36]. The statistical model included diarrhoea at the pen or individual level as the response variable, diet and week as fixed factors, and batch and individual ID (only for individual diarrhoea) as random factors, considering that the measurements were repeated over time. The potential interaction effects of diet and week were examined via chi-square tests with the full model. Whenever an interaction or fixed effect was deemed non-significant (*p* > 0.05), it was removed from the model, which was then re-analysed. In case any significant effect of treatment, week, or their interaction was detected, pairwise comparisons were performed using Tukey’s test for the significant outcome(s).

Average daily gain (ADG) was calculated based on the initial and final body weight of 127 individuals, excluding the animals that died during the weaning period. A linear mixed-effects model (LMM) was used from the lme4 package v1.1-35.3 [37], including ADG as the response variable, diet and feed intake as fixed factors, and batch as a random factor. The individual was the experimental unit. The potential interaction effects of diet, feed intake, and initial weight were examined via ANOVA with the full model. Whenever an interaction or fixed effect was deemed non-significant (*p* > 0.05), it was removed from the model, which was then re-analysed. The normal distribution of the residuals of the fitted model was checked. If it did not follow a normal distribution, our response was transformed, and the process was repeated. In the event that any significant effect of diet, feed intake, or their interaction was detected, pairwise comparisons were performed using Tukey’s test for the significant outcome(s).

Given that residual behavioural data were not normally distributed, GLMM models were used from the lme4 package [37]. The models included each scan and focal behaviour as the response variables, diet and week as fixed factors, batch as a random factor, and considering that measurements were repeated over time. The pen was the experimental unit. The potential interaction effects of diet and week were examined via chi-square tests with the full model. Whenever an interaction or fixed effect was deemed non-significant (*p* > 0.05), it was removed from the model, which was then re-analysed. If any significant effect of treatment, week, or their interaction was detected, pairwise comparisons were performed using Tukey’s test for the significant outcome(s). A selective approach was adopted for additional health parameters, given their low incidence, where only those with >40% of non-zero scores were selected. When applying these criteria, sneezing was the only additional health parameter included for statistical analysis. The analysis of sneezing was the same as the methods used for the behavioural data.

The microeco package [38] was used to plot the taxonomic composition and compare the diversity index across conditions. The α-diversity was evaluated with the Shannon index, and the β-diversity was assessed using the Whittaker index. The differential abundance analysis to compare the genus abundance table across dietary interventions was carried out with the ANCOM-BC2 package v2.6.0 [39]. Subsequently, multiple pairwise comparisons were performed among the three groups while controlling the overall mFDR at 0.1.

## 3. Results

### 3.1. Diarrhoea at the Individual and Pen Level

**Figure 1:**
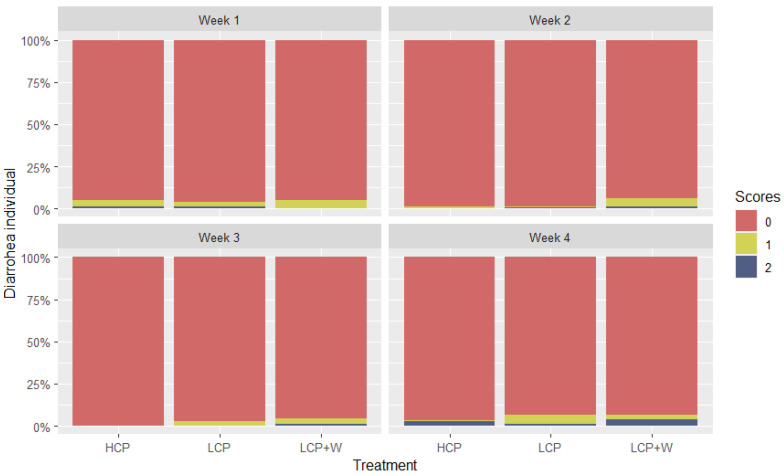
Percentage of the diarrhoea scores at the individual level (0 = no diarrhoea; 1 = mild diarrhoea; 2 = severe diarrhoea) of the three diets for each of the four assessed weeks. On week 1, 9.1% of the individuals showed mild and 1.5% severe diarrhoea; on week 2, 5.3% showed mild and 2.3% severe diarrhoea; on week 3, 6.1% showed mild and 1.5% severe diarrhoea; and on week 4, 8.3% showed mild and 9.1% severe diarrhoea.

Regarding diarrhoea at the individual level, of the 134 individuals included in this study, 7.4% showed mild diarrhoea and 3.8% had severe diarrhoea on the assessment days. In the HCP diet, 3.5% showed mild diarrhoea and 2.9% severe diarrhoea; in the LCP diet, 8.8% of the individuals showed mild diarrhoea and 2.3% severe diarrhoea; and in the LCP+W diet, 9.8% showed mild diarrhoea and 5.8% severe diarrhoea on the assessment days. The percentages of diarrohea scores at the individual level per week are shown in Figure 1.

The results of the model with diarrhoea at individual and pen levels as a response did not show a significant interaction effect between diet and feed intake or diet and initial weight (*p* > 0.05). Thus, we proceeded with the analysis of the models without interaction effects. The results of the model with diarrhoea at the individual level did not show a significant diet (X^2^ = 4.26, df = 2, *p* = 0.118), week (X^2^ = 4.12, df = 3, *p* = 0.246), or diet and week interaction effect (X^2^ = 4.21, df = 2, *p* = 0.1218). The results of the model with diarrhoea at pen level did not show a significant diet (X^2^ = 1.39, df = 2, *p* = 0.4968) or week (X^2^ = 2.43, df = 3, *p* = 0.1189) effect.

### 3.2. Health Parameters

From the additional health parameters studied, only sneezing presented >40% of non-zero scores. The results of the LMM with sneezing as a response did not show any significant diet differences (F2,57 = 0.32, *p* = 0.7259). Regarding tail lesions, 0.58% of HCP piglets, 1.75% of LCP, and 0% of LCP+W presented superficial tail biting (score 1). Of the 134 individuals included, 7 died during the study: 1 in the HCP, 3 in the LCP, and 3 in the LCP+W treatment. Thus, the overall mortality in the weaning phase was 5.2%.

### 3.3. Average Daily Gain and Consumption

The mean initial and final body weight and ADG per treatment, as well as the average solid feed intake consumed during the weaning period were calculated per diet (see Table 4 below).

**Figure 2:**
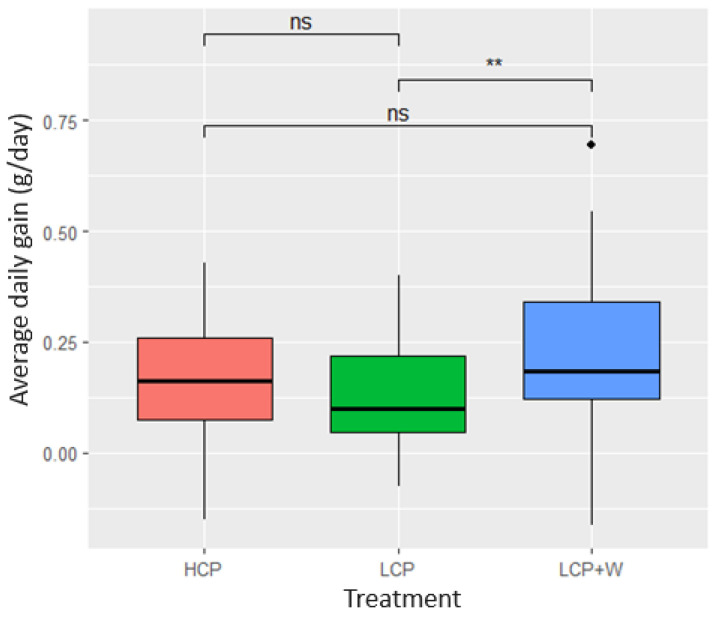
Boxplot of the differences in ADG among treatments and their pairwise comparisons. *p* > 0.05 (ns); *p* < 0.001 (**).

The results of the LMM with ADG as a response did not show a significant interaction effect between diet and feed intake or diet and initial weight (*p* > 0.05). Thus, we proceeded with the analysis of the model without interaction effects. Significant differences in ADG between diets were shown (F2,126 = 9.88, *p* = 0.0001), but there was no significant relationship between feed intake and ADG (F1,8 = 2.38, *p* = 0.1601). The results of Tukey’s test for diet showed that LCP+W piglets presented a significantly higher ADG (*p* = 0.0004) compared to LCP treatment (Figure 2).

### 3.4. Behavioural Parameters

**Figure 3:**
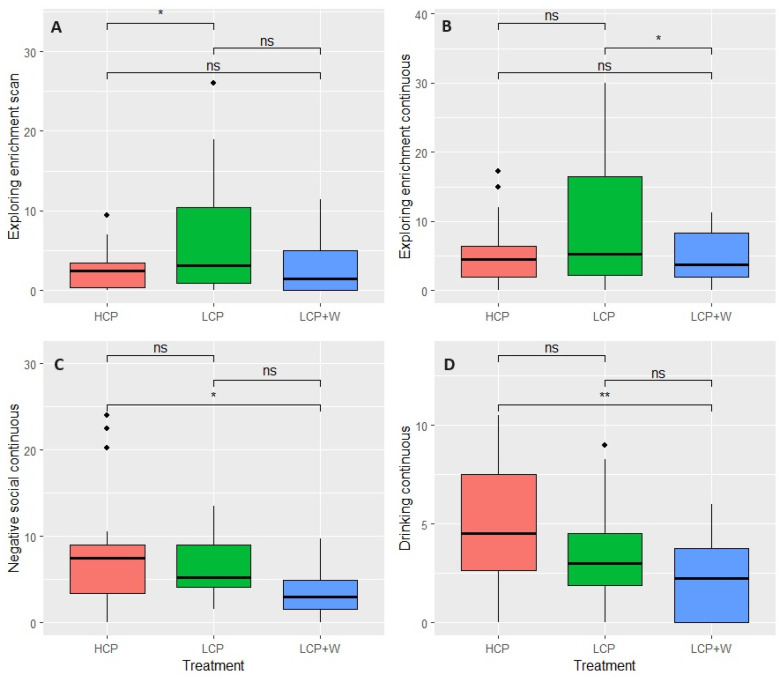
Boxplots of the behaviour differences among diets. The plot shows only the behaviours with significant diet differences and their pairwise comparisons: exploring enrichment scan (**A**), exploring enrichment continuous (**B**), negative social continuous (**C**), and drinking continuous (**D**). *p* > 0.05 (ns); *p* < 0.05 (*); *p* < 0.001 (**).

The results of the GLMM with each of the behaviours as responses did not show a significant interaction effect between diet and feed intake or diet and initial weight (*p* > 0.05). Thus, we proceeded with the analysis of the model without interaction effects. The results of the GLMM showed significant diet differences in exploring enrichment evaluated with scan sampling (X^2^ = 8.50, df = 2, *p* = 0.014, Figure 3A); and for the continuous observations in negative social (X^2^ = 9.18, df = 2, *p* = 0.011, Figure 3C), exploring enrichment (X^2^ = 8.24, df = 2, *p* = 0.016, Figure 3B), and drinking (X^2^ = 12.47, df = 2, *p* = 0.001, Figure 3D) behaviours. The results of Tukey’s tests showed that LCP piglets performed a significantly higher percentage of enrichment exploration in the scans compared to HCP (*p* = 0.024, Figure 3A) and compared to LCP+W in the continuous observations (*p* = 0.030, Figure 3B). HCP piglets performed a significantly higher percentage of socially negative behaviours (*p* = 0.012, Figure 3C) and drank significantly more (*p* = 0.002, Figure 3D) during continuous observations compared to LCP+W piglets. No other significant differences in behaviour, either during the scan or continuous observations between diets, were found. The results of the GLMM also showed significant week differences for the following behaviours in the scan sampling: pen investigation (X^2^ = 12.84, df = 3, *p* = 0.004), which was higher in visit 1 than in visit 2 (*p* = 0.004); exploring enrichment (X^2^ = 11.00, df = 3, *p* = 0.011), which was higher in visit 1 than visit 2 (*p* = 0.009); resting (X^2^ = 9.61, df = 3, *p* = 0.022), which was higher in visit 2 than visit 4 (*p* = 0.020); and other behaviours (X^2^ = 10.52, df = 3, *p* = 0.014), which was lower in visit 1 than visit 4 (*p* = 0.012). For the continuous observations, positive social behaviour (X^2^ = 8.91, df = 3, *p* = 0.030) was higher in visit 1 than in visit 2 (*p* = 0.022), and exploring enrichment (X^2^ = 11.44, df = 3, *p* = 0.009) was higher in visit 1 than visit 2 (*p* = 0.010). No other significant differences in behaviour, either during the scan or during continuous observations between weeks, were found. The mean and standard deviation values for each of the scans and continuous behaviours per diet and week are shown in the Supplementary Materials section (Tables S1 and S2).

### 3.5. Intestinal Microbiota Composition

**Figure 4:**
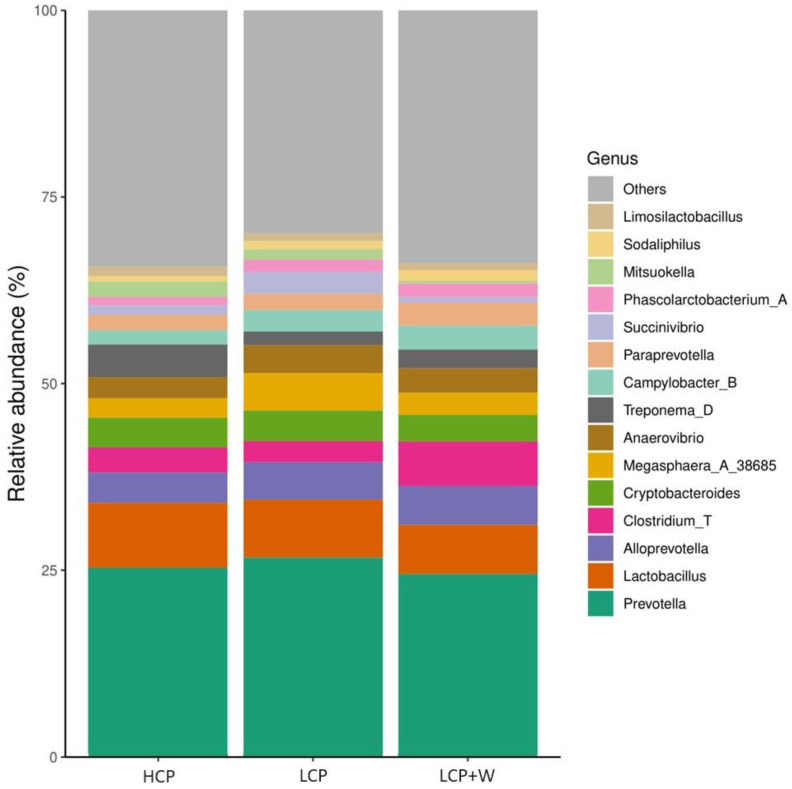
Mean of the relative abundance shown on the genus taxonomy level in the three studied diets.

At the phylum level, sixteen phyla were detected in the three studied diets. The most abundant were *Bacteroidetes* (43.3%) and *Firmicutes* (42.3%), followed by *Proteobacteria* (6.7%) and *Spirochaetes* (4%), while the most abundant genera were *Prevotella*, *Lactobacillus*, *Alloprevotella*, and *Clostridium* (Figure 4).

**Figure 5:**
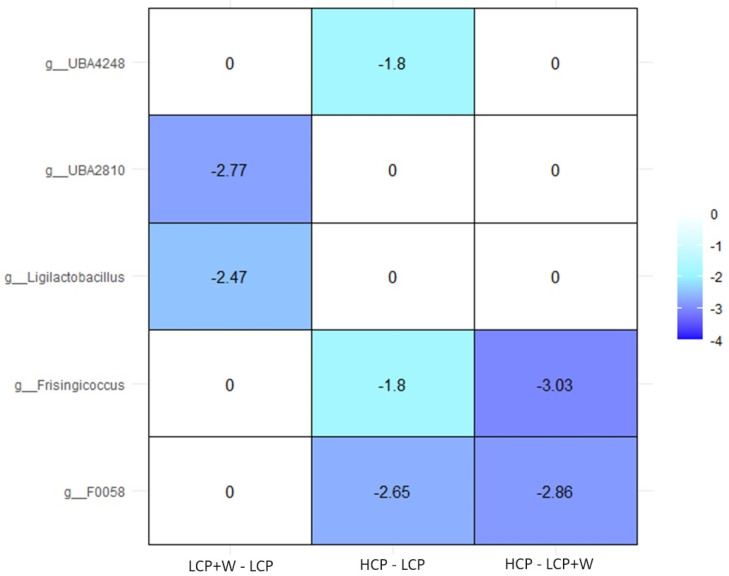
Heatmap of ANCOM-BC2 pairwise analysis for the effect of the different diets. The *X*-axis represents the specific pairwise comparisons, and the *Y*-axis displays the significant genus identified by ANCOM -BC2. Each cell in the heatmap is colour-coded, with blue representing reduced abundance, white representing non-differences, and the numbers on each cell indicating the log fold change. The Holm-Bonferroni method was utilised to correct for multiple tests.

Our analysis revealed differences in only a few genera. Compared to LCP, the LCP+W diet reduced the abundance of *UAB2810* and *Ligilactobacillus*. Meanwhile, LCP+W resulted in an increase in *Frisingicoccus* and *F0058* members of the family Lachnospiraceae and Paludibacteraceae, respectively, compared to both LCP and HCP diets (Table 5 and Figure 5).

## 4. Discussion

### 4.1. Diarrhoea at Individual and Pen Level

The results of this study showed that diet had no effect on diarrhoea incidence; neither LCP nor LCP+W diets decreased the incidence of PWD compared to the HCP diet. On the contrary, several studies concluded that a decreased crude protein content diet reduced the incidence of PWD in piglets [14,15,16,40]. These studies were conducted on conventional farms, where the need for high productivity leads farmers to feed the animals diets containing high amounts of crude protein, ranging from 210 to 250 g/kg [13]. In contrast, the crude protein content given to post-weaning piglets in organic farming is much lower than in conventional ones, usually inferior to 180 g/kg, given the nature of organic farming and the lack of protein sources available [41]. Thus, the reduction in the crude protein content may only have a marked effect on diarrhoea when comparing very high crude protein diets (210–250 g/kg) with lower crude protein content diets (<180 g/kg), but not when reducing the crude protein content from 178 g/kg to 168 g/kg, as was the case in this study. This could partially explain our results, as this crude protein reduction is not enough to have any significant effect on diarrhoea incidence. For this reason, a further reduction in the crude protein content in post-weaning organic diets may not be an appropriate approach to reducing PWD, and other nutritional strategies should be explored.

Diarrhoea incidence is not only influenced by the crude protein content of the diet but also by its composition. Some researchers suggested that the use of animal protein sources is preferred over plant-based ones, given their optimal composition and higher digestibility, which benefits the health of post-weaned piglets [20,42]. In addition, the use of lactic acid, such as that contained in liquid whey, reduced the incidence of PWD in some studies [25,43]. This was not the case in our study, where neither the HCP diet containing milk powder nor the diet supplemented with liquid whey showed a difference in diarrhoea incidence compared with the plant-based LCP diet. In the present study, the overall diarrhoea incidence was 11.2% of the individuals showing mild or severe diarrhoea. This incidence was low compared with studies conducted in organic farms that reported a diarrhoea incidence of 21.2–44.4% on the peak of PWD at 5 days after weaning [44]. The low incidence of diarrhoea found in this study may partially explain why we did not find significant diet and time differences and could be attributed to an exhaustive control of risk factors in the studied farm that includes adequate hygiene, a delayed weaning age, an optimal temperature and ventilation, and a low stocking density [4]. Still, an appropriate post-weaning diet could be an optimal way to reduce the risk of PWD when other factors cannot be controlled.

### 4.2. Health Parameters

In the present study, we detected a low incidence of all of the additional health parameters analysed. The only health parameter recurrent enough to be analysed was sneezing, but it did not show significant differences among diets. Studies comparing the health status of animals in organic versus conventional farming showed no differences between them, not ensuring organic systems had higher health for the animals [3]. This is explained by the restricted use of medicines as a preventive measure in organic farming, which forces farmers to find alternative preventive strategies to reduce the risk of certain diseases developing [1]. Despite this, our results show a positive health state of the studied animals with regard to the health parameters assessed in this study. An important remark along that line is that no negative impact of any of the diets on tail damage was found in our study, reinforcing previous studies that indicate that if amino acid balance is well achieved in diets containing lower overall protein content, damaging behaviours may not arise to a greater extent compared to control diets [19]. Data concerning the prevalence of the studied health parameters in organic farming are scarce [45], so further research should be conducted along that line in organic pig production.

### 4.3. Average Daily Gain and Consumption

Although pointed out as a useful strategy to decrease PWD, several studies found that diets with a lower protein content showed lower growth rates of piglets weaned at 21–28 days compared with high protein content diets [15,16,18]. In this study, though, the high and low crude protein diets did not show a significant difference in the ADG of piglets. This could also be explained by a similar hypothesis to those presented for the lack of differences with regards to diarrhoea, being that the protein content was already low in both diets compared with similar studies conducted in conventional farming. Several studies have demonstrated that feeding a diet with a low protein content may not compromise growth if supplemented with high-quality energy-content supplements [38,46]. For example, some studies found that the weight loss caused by low-protein diets could be supplemented by adding whey, which enhances the piglets’ ADG [23]. Similarly, our results showed that the use of liquid whey as a supplement to a low-protein-content diet increases the ADG of post-weaned piglets. Liquid whey meets the requirements to be an optimal protein supplement source, given its high digestibility, energy content, and pleasant taste [47]. It also contains a high amount of lactose, which is thought to be an essential nutrient to help post-weaned piglets achieve their maximal performance [48].

During the first post-weaning days, the growth rates of piglets decrease because their voluntary feed and water intake are suddenly reduced, even when the weaning age is delayed [49]. As some studies have demonstrated, the use of whey increases the daily feed intake of piglets due to its pleasant flavour [23]. Despite the fact that the animals in the three diets consumed similar amounts of solid feed, piglets in the LCP+W diet consumed an extra 13.3 L/individual of liquid whey in addition to the solid feed. Thus, the supplement of liquid whey did not reduce the solid feed intake of these animals but increased their overall feed intake, which explains the increased ADG of the piglets supplemented with liquid whey. Thus, liquid whey is a nutritional supplement that the piglets can recognise and are motivated to consume, which allows them to recover voluntary feed intake soon after weaning, avoiding their reduced growth rate during the first days post-weaning.

In addition to the benefits for the piglets, the use of liquid whey could also be an important nutritional implementation for organic farmers. The lower productivity of organic farming compared with conventional systems is caused by the limited availability of high-quality feedstuffs with high amino acid content and high digestibility [3]. Liquid whey meets these nutritional characteristics, as well as being in line with the requirements of organic farming and having a low price. On the contrary, other animal-origin supplements, such as milk powder, may not meet organic standards and have a high price [50]. Thus, the use of liquid whey would reduce the production costs of organic farming, increasing its benefits as well as improving their self-sufficiency rate and solving the issue of the lack of protein sources in organic farming. The addition of liquid whey to the piglets’ diet would also give use to this by-product of the cheese industry, reducing its negative environmental impact and also decreasing the costs of its disposal [51]. Thus, liquid whey is a potential nutritional supplement for post-weaned piglets that could enhance their growth rates and increase productivity in organic farm systems.

### 4.4. Behavioural Parameters

From the behaviours studied, only exploration of the enrichment material, social negative interactions, and drinking behaviours appeared to differ among diets. The increased explorative behaviour in the LCP compared with HCP and LCP+W diets may be explained by the so-called “appetitive behaviour,” which is motivated by a need to consume feed when the animal is hungry or lacking nutrients in sufficient amounts [52]. This could indicate that a plant-based, low crude protein diet might be insufficient for post-weaned piglets, motivating them to search for additional nutrients through foraging the straw. On the contrary, there was no significant effect of the diets on the increase in oral behaviours directed either to the tail or ears of the pigs, which is in line with the low incidence of tail lesions and the lack of differences in any of the treatments. Tail biting has been said to have a multifactorial origin, and the low incidence on this farm could be attributed previously to the control of risk factors like health, stocking density, delayed weaning age, the provision of adequate enrichment material, and climate conditions [53]. A reduced number of negative social behaviours were seen in the LCP+W compared with the HCP diet, which could be related to the fact that the LCP+W pen had an extra feeder containing liquid whey, while in the HCP and LCP diets, the pen had a single feeder. The presence of two feeders allowed the animals to have an adequate feed space, reducing the competition among them and decreasing agonistic behaviours and negative interactions [54]. In the present study, drinking behaviour was reduced in the LCP+W compared with the HCP diet. In the LCP+W diet, the water that the piglets drank was not only supplied by the water dispensers but also by the water content of the liquid whey, which may reduce their drinking behaviour due to the fact that the water requirement was already provided by the consumption of liquid whey. Water intake is an important factor during the first days post-weaning, when there is a reduced voluntary water intake [9] that can be provoked by the change in the type of water dispenser or drinker, which the piglets need to get used to [55]. Thus, the intake of water already provided by liquid whey may also have a positive impact by increasing the total voluntary water intake and reducing the impact of the adaptation to new drinking systems when only supplied by water dispensers.

In addition to the differences between diets, we found several significant week differences, with the most notorious being a decrease in “other” behaviours observed by scan in week 1 compared to week 4. These “other” behaviours included eating and drinking in the scans, which may indicate that during the first week after weaning, the piglets may have reduced the time they spent at the feeder and water supplies, as seen in other studies [9]. Despite this, we did not find week-long differences in drinking and eating when performing continuous observations. Moreover, during the first week after weaning, the animals presented a higher percentage of pen investigation and enrichment exploration behaviours, which could be due to the piglets being introduced to a new environment [56]. Piglets also presented more positive social behaviours just after weaning than in the second week, which could be due to a change in the social group after mixing and their need to establish new social bonds [57].

### 4.5. Intestinal Microbiota Composition

At the phylum level, the most dominant groups were *Bacteroidetes* (43.3%) and *Firmicutes* (42.3%), which is in agreement with other studies in which these two phyla have been detected as the most dominant ones in porcine intestinal microbiota, accounting for more than 80% of the faecal microbiota [58]. *Proteobacteria* was the third most abundant phylum in our study (6.7%), which includes a great variety of opportunistic microorganisms such as *E. coli.* Even with this relatively high abundance, the presence of *Proteobacteria* does not indicate impaired health because these bacteria are seen both in healthy and diseased piglets [59].

Some authors stated that the source of the dietary content is one of the most important factors affecting intestinal microbiota diversity [24]. In the present study, the results from the differential abundance analysis showed that, compared to the LCP and HCP diets, the LCP+W diet increased the abundance of *Frisingicoccus* and the *F0058* genus. *Frisingicoccus* is a butyrate-producing bacteria member of the family Lachnospiraceae [60]. Interestingly, *Frisingicoccus* has been reported to be associated with gut health in humans, and in agreement with our findings, it is positively linked with body weights in pigs [61] and with high faecal scores, being depleted in the gut of dairy cows with diarrhoea [62]. On the other hand, *F0058* is a member of the Paludibacteraceae family, and previous studies have reported that members of this family were linked to the fermentation of wheat bran fibre in sows [63] but also more abundant in pigs under a low-protein diet supplemented with branched-chain amino acids [64]. To be noted, in this study, the increase in the abundance of Paludibacter was followed by a decrease in opportunistic microorganisms from the Streptococcaceae and Pseudomonadaceae families. Thus, the increased abundance of the genus *Frisingicoccus* in the diet supplemented with whey could cause positive effects on the piglets’ intestinal microbiota, explaining the increased ADG of piglets supplemented with liquid whey found in the present study.

## 5. Conclusions

Given its restrictions and the great variability among farms, ensuring health standards on organic farms is a challenge that is sometimes difficult to achieve. For this reason, preventive strategies such as an adequate diet are the best approach to dealing with animal health and welfare in organic systems. The use of dietary supplements that meet organic standards, such as liquid whey, is a promising approach. Even though neither a reduced crude protein content nor liquid whey supplementation showed an effect on PWD, liquid whey supplemented with a plant-based organic diet improved the growth rate of post-weaned piglets, as well as having a positive effect on their intestinal microbiota composition by increasing the abundance of the genera *Frisingicoccus.* The high lactose content of liquid whey makes it an attractive feedstuff for piglets, as well as being a high-quality protein and energy source. A lower incidence of negative social behaviour and a higher percentage of drinking were also seen in the diet supplemented with liquid whey, quite likely associated with the fact that an additional feed trough was available for the animals. No negative impact of the diets was found on damaging behaviours such as tail or ear biting or tail lesions. Thus, liquid whey could be an optimal addition to the post-weaned organic piglet diet, given its benefits to the animals. This addition would not only be beneficial to the animals but could also help organic farms supply the lack of protein sources available in organic systems, increasing their efficiency. In addition, liquid whey perfectly fits organic standards, making it easier to achieve the self-sufficiency goal of organic systems.

## Figures and Tables

**Table 1:** Energy content (kcal/kg) and nutritional composition (% crude content) of the three different diets used: HCP, LCP, and LCP+W.

	HCP	LCP	LCP+W
Energy (kcal/Kg)	3400	2900	2900 + 240 *
Protein	17.80	16.80	17.67
Lactose	-	-	4.07
Fiber	2.40	4.70	4.70
Oil and fat	4.60	3.10	4.23
Ashes	4.80	6.20	6.71
Lysine	0.57	0.81	0.81
Methionine	0.32	0.26	0.26
Calcium	0.83	1.12	1.12
Sodium	0.20	0.21	0.26
Phosphorus	0.65	0.70	0.70
Salt	-	-	0.10

* Net energy of whey was not provided in the compositional analysis, so the value is based on [28].

**Table 2:** Additional health parameters were assessed.

Health Parameter	Description/Scores	Individual or Group Level
Coughing	The pig cough	Group
Sneezing	The pig sneeze	Group
Pumping	The pig’s breathing is heavy and laboured and its chest is raising and falling with each breath	Group
Huddling	A pig is lying with more than half of its body in contact with another pig	Group
Shivering	A slow and irregular vibration of any body part or the whole body	Group
Panting	Breathing rapidly in short gasps carried out with the mouth	Group
Body condition score	0 = good condition; 1 = lean animal: visible spin, hip, or pin bones	Individual
Skin condition	0 = no skin inflammation or discolouration; 1 = up to 10% of the skin is inflamed, discoloured, or spotted; 2 = more than 10% of the skin is inflamed, discoloured, or spotted	Individual
Tail lesions	0 = no lesions on the tail; 1= superficial biting but no fresh blood or swelling; 2 = fresh blood and swelling	Individual
Manure on the body	0 = up to 20% of the body is soiled; 1= more than 20% but less than 50% of the body is soiled; 2 = over 50% of the body is soled	Individual

**Table 3:** Ethogram of the behavioural parameters assessed.

Behaviour	Description	Sampling Method
Positive social	An affiliative interaction including sniffing, nosing, licking, or any social behaviour without a response from the receiver animal	Scan and continuous
Negative social	An aggressive interaction including biting, knocking, or any social behaviour with a flight or reaction response from the receiver animal	Scan and continuous
Exploring enrichment	Play or investigate by sniffing, nosing, licking, or chewing the straw or other enrichment material	Scan and continuous
Pen investigation	Sniffing, nosing, licking, or chewing all features of the pen	Scan
Resting	Animals lying ventrally or laterally and not showing any exploratory, positive, or negative social behaviour	Scan
Other	Other active behaviours, such as eating, drinking, or air sniffing	Scan
Eating	Ingestion of feed from the feeder	Continuous
Drinking	Ingestion of water from the drinking supply	Continuous
Tail biting	Having the tail of another pig in its mouth biting, chewing, or pulling it	Continuous
Ear biting	Having the ear of another pig in its mouth biting, chewing, or pulling it	Continuous

**Table 4:** Mean (SD) of initial body weight, final body weight, average daily gain, and feed intake per diet.

Diet	Initial BW (kg)	Final BW (kg)	ADG (g/day)	Feed Intake (kg/Individual) *
HCP (*n* = 44)	12.88 (3.49)	16.39 (4.99)	162.92 (142.18)	15.1
LCP (*n* = 43)	12.56 (3.72)	15.98 (5.08)	131.87 (112.65)	14.1
LCP+W (*n* = 47)	12.04 (4.11)	16.87 (6.39)	226.21 (172.44)	13.6 + 13.3 L/individual of liquid whey

* Estimated by dividing pen intake/number of pigs.

**Table 5:** Resume the differential abundance genus, and Figure 5 shows the log fold change in the pairwise comparisons among diets.

GENUS	W	PVAL	QVAL
UBA2810	39	0.00001	0.002
FRISINGICOCCUS	32	0.00002	0.004
F0058	26	0.00002	0.004
LIGILACTOBACILLUS	25	0.00020	0.050

## Data Availability

The raw data supporting the conclusions of this article will be made available by the authors upon request.

## Supplementary Materials

The following supporting information can be downloaded at: https://www.mdpi.com/article/10.3390/ani14121730/s1; Table S1: Mean (SD) for each of the scan behaviours showing the overall by treatment and per week; Table S2: Mean (SD) for each of the continuous behaviours showing the overall by treatment and per week.
