# Supplementary material for: Impact of Nutritional Strategies to Prevent Post-Weaning Diarrhoea on Performance, Behaviour, and Microbiota in Piglets from Organic Farming
Source: Animals (Basel). 2024 Jun 8;14(12):1730. doi: 10.3390/ani14121730 (PMC11200382; doi:10.3390/ani14121730)
Supplement: Supplementary file 1 [file animals-14-01730-s001.zip › animals-3013615-supplementary.pdf]

Figure S1. Mean (SD) for each of the scan behaviours showing the overall by treatment and per each week.

Week	Positive social			Negative social			Exploring enrichment			Pen investigation			Resting			Other		
	HCP	LCP	LCP+W	HCP	LCP	LCP+W	HCP	LCP	LCP+W	HCP	LCP	LCP+W	HCP	LCP	LCP+W	HCP	LCP	LCP+W
Overall	2.06 (1.88)	2.18 (2.36)	1.91 (2.32)	1.34 (1.32)	0.97 (1.61)	0.53 (0.56)	2.52 (2.59)	6.55 (7.37)	3.15 (3.58)	13.34 (12.00)	12.46 (10.83)	9.48 (8.94)	50.61 (22.62)	51.58 (19.22)	60.78 (18.48)	30.10 (16.82)	26.22 (12.10)	24.23 (11.33)
Week 1	2.66 (2.07)	1.48 (2.71)	2.54 (3.49)	0.33 (0.74)	1.02 (1.28)	0.89 (0.59)	4.00 (3.47)	10.54 (9.44)	6.45 (3.68)	19.39 (18.07)	16.20 (15.66)	15.87 (12.39)	48.83 (30.03)	53.55 (24.88)	56.09 (25.37)	24.77 (10.18)	17.17 (14.14)	18.12 (11.47)
Week 2	1.47 (1.79)	1.29 (1.77)	1.45 (2.28)	1.34 (1.14)	0.44 (0.63)	0.00 (0.00)	1.18 (1.62)	2.41 (4.33)	0.83 (1.24)	7.88 (10.58)	5.04 (5.07)	5.76 (6.50)	56.83 (28.61)	62.81 (15.90)	74.90 (13.84)	31.26 (21.85)	27.99 (14.36)	17.44 (9.62)
Week 3	3.25 (1.80)	2.61 (2.76)	1.26 (1.72)	1.90 (1.32)	0.00 (0.00)	1.40 (0.76)	3.00 (2.82)	6.71 (6.45)	4.54 (2.57)	15.77 (8.84)	12.84 (10.18)	6.74 (7.95)	47.41 (14.87)	51.41 (16.55)	60.93 (14.37)	28.63 (13.95)	26.41 (7.35)	27.70 (6.20)
Week 4	0.55 (0.64)	3.63 (2.01)	2.49 (1.74)	1.90 (1.73)	2.80 (2.51)	0.44 (0.53)	1.77 (1.57)	6.55 (8.44)	2.66 (4.66)	9.58 (6.02)	16.59 (8.16)	9.55 (5.56)	49.04 (19.80)	35.30 (10.92)	48.81 (10.04)	37.12 (23.31)	35.09 (4.22)	36.01 (8.69)

Figure S2. Mean (SD) for each of the continuous behaviours showing the overall by treatment and per each week.

Week	Positive social			Negative social			Exploring enrichment			Eating			Drinking			Tail or ear biting		
	HCP	LCP	LCP+W	HCP	LCP	LCP+W	HCP	LCP	LCP+W	HCP	LCP	LCP+W	HCP	LCP	LCP+W	HCP	LCP	LCP+W
Overall	7.34 (4.35)	5.13 (3.76)	5.96 (4.21)	8.01 (7.05)	6.35 (3.37)	3.55 (2.71)	5.32 (4.95)	9.71 (9.39)	4.73 (3.70)	13.93 (13.30)	15.75 (8.41)	11.09 (7.26)	5.05 (3.20)	3.43 (2.37)	2.21 (2.17)	2.21 (2.66)	2.56 (2.25)	1.18 (1.62)
Week 1	9.30 (3.20)	6.60 (5.69)	8.10 (5.59)	7.35 (7.69)	5.25 (3.00)	4.35 (3.57)	8.70 (5.55)	13.35 (8.30)	7.80 (3.77)	6.60 (5.17)	9.60 (10.19)	10.95 (8.72)	4.50 (2.05)	3.01 (3.09)	2.40 (2.56)	1.35 (1.86)	3.60 (3.41)	1.05 (1.46)
Week 2	5.10 (2.97)	4.05 (4.39)	3.30 (2.40)	5.85 (3.76)	5.85 (4.63)	2.40 (0.97)	2.85 (2.50)	4.65 (6.88)	1.35 (1.86)	19.65 (14.40)	21.00 (7.32)	8.25 (7.77)	5.40 (4.22)	3.30 (1.55)	2.70 (2.28)	1.05 (1.55)	1.35 (1.62)	1.05 (1.46)
Week 3	7.95 (6.76)	5.25 (2.37)	4.50 (3.47)	9.15 (8.76)	6.90 (3.49)	2.55 (2.22)	5.70 (6.55)	12.60 (12.50)	4.80 (3.29)	13.05 (4.67)	17.25 (6.00)	12.45 (6.06)	4.95 (3.50)	2.55 (1.95)	1.95 (2.57)	3.75 (4.30)	3.00 (1.98)	0.45 (1.00)
Week 4	6.93 (3.43)	4.50 (1.50)	8.43 (3.14)	10.12 (9.05)	7.68 (2.47)	5.25 (3.24)	3.75 (2.80)	7.87 (9.09)	5.06 (3.08)	17.06 (23.72)	15.00 (7.27)	13.12 (7.99)	5.43 (3.84)	5.25 (2.59)	1.68 (1.66)	2.81 (1.41)	2.25 (1.22)	2.43 (2.40)
